# Transcatheter stenting of superior vena cava to treat postoperative SVC syndrome in a child: a case report

**DOI:** 10.1186/s43044-024-00547-6

**Published:** 2024-08-29

**Authors:** Somrita Laha, Debasree Gangopadhyay, Mahua Roy, Jayitri Mazumdar, Mrinalendu Das, Patralekha Das

**Affiliations:** 1 Department of Pediatric Cardiology, NH-Rabindranath Tagore International Institute of Cardiac Sciences, Premises No: 1489, Mukundapur Main Road, 124, Eastern Metropolitan Bypass, Mukundapur, Kolkata, West Bengal 700099 India; 2 Department of Cardiothoracic and Vascular Surgery, NH-Rabindranath Tagore International Institute of Cardiac Sciences, Kolkata, India

**Keywords:** Total anomalous pulmonary venous connection (TAPVC), Iatrogenic superior vena cava (SVC) obstruction, Balloon angioplasty of SVC

## Abstract

**Background:**

Superior vena cava (SVC) obstruction leading to SVC syndrome is an uncommon but potential complication of cardiac surgeries that involve dissection and anastomosis around the great vein. We present a case of iatrogenic SVC obstruction that was initially treated with transcatheter balloon angioplasty, which provided temporary relief, and ultimately resolved by stenting the affected segment.

**Case presentation:**

The index case underwent total anomalous pulmonary venous connection (TAPVC) repair and presented 3 months after surgery with features of SVC obstruction. Initially, transcatheter balloon angioplasty was performed, providing relief from the obstruction; however, the condition recurred within one month. Finally, the patient was treated with percutaneous stenting of superior vena cava, through femoral venous route, using 8 mm × 30 mm balloon-expandable bare metal stent (Formula 418, Cook Medical, Bloomington, IN). Remarkable relief of obstruction was established with decrease in mean gradient across SVC–right atrium junction to 2 mm Hg (from 12 mm Hg before balloon angioplasty and 18 mm Hg before stenting).

**Conclusion:**

Percutaneous treatment for iatrogenic SVC obstruction developing after cardiac surgery appears to be effective. Close monitoring is required in the postoperative period for early diagnosis and timely intervention.

## Background

Globally, intrathoracic malignancy remains the most common cause of superior vena cava (SVC) obstruction leading to SVC syndrome. However, iatrogenic SVC obstruction can also occur as a complication of cardiac surgeries involving the dissection and anastomosis of the great vein [1]. This postoperative complication can be seen after repair of supracardiac, cardiac or mixed TAPVC (total anomalous pulmonary venous connection) [2]. Obstruction of venous drainage of upper body due to SVC obstruction leads to venous hypertension and thereby engorgement of veins and swelling of upper body [2]. It may even lead to serious consequences like raised intracranial pressure, airway blockage and respiratory distress [3].

## Case presentation

A 7-month-old boy, weighing 5.7 kg, underwent rerouting of total anomalous pulmonary venous connection (TAPVC) with common chamber draining at the junction of right superior vena cava (RSVC) and right atrium (RA). On cardiopulmonary bypass, the atrial septal defect (ASD) was enlarged and closed with a glutaraldehyde-treated autologous pericardial patch, thereby incorporating pulmonary venous drainage to left atrium (LA), however, the anterior wall of SVC was not augmented with patch. The immediate postoperative period was uneventful. Pre-discharge echocardiography showed unobstructed pulmonary venous return to LA, with only mild flow turbulence at lower end of RSVC without any anatomical obstruction, and a mean gradient of 1 mm Hg. On 6-week follow-up, the patient was doing well without any concern regarding cardiac status.

**Fig. 1:**
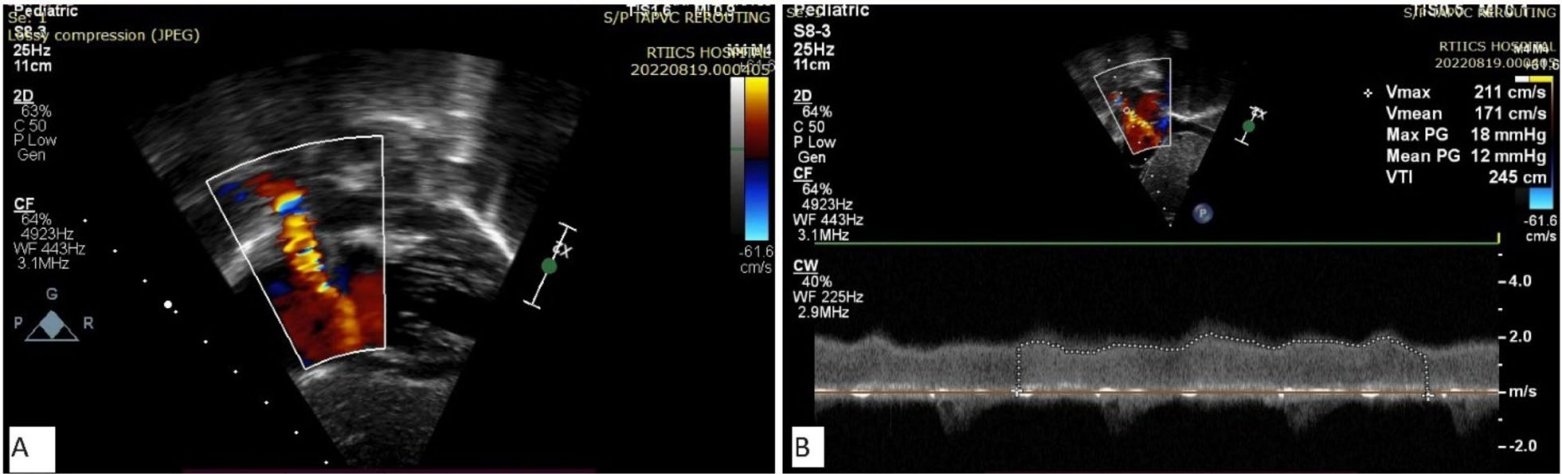
Subcostal bicaval view showing flow aliasing across SVC–RA junction (**A**) with mean gradient 12 mm Hg (**B**)

**Fig. 2:**
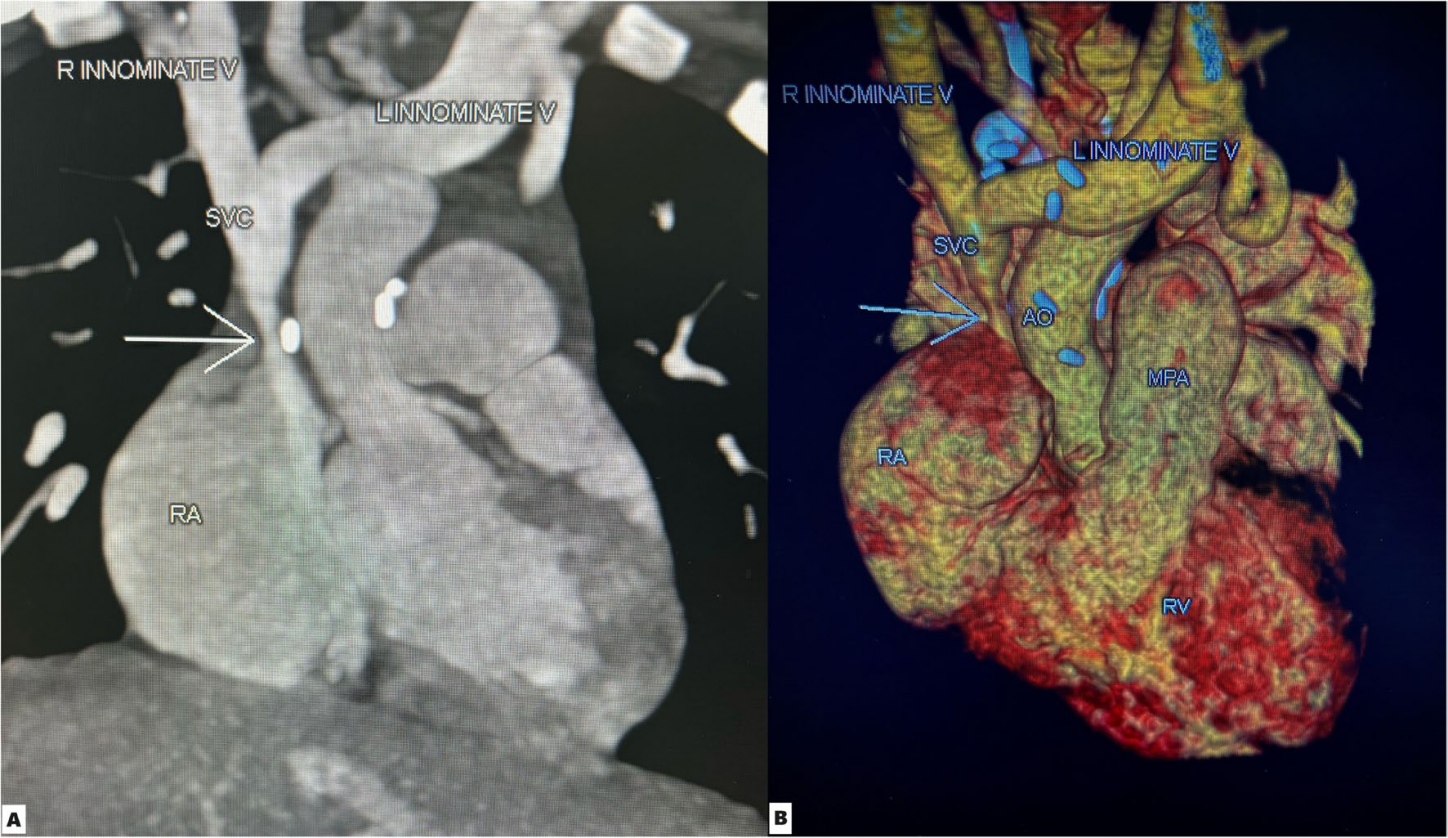
CT angiogram images of coronal view (**A**) and 3D reconstruction (**B**) showing narrowing at lower end of SVC

However, on 3-month follow-up (weight 7.4 kg), the patient had started developing symptoms of superior vena cava (SVC) syndrome: puffiness of face and eyelids, engorged neck veins and mild respiratory distress. Echocardiography showed narrowed lower end of RSVC with significant flow aliasing and mean gradient of 12 mm Hg (Fig. 1). Pulmonary venous drainage was normal, atria septal defect (ASD) patch was intact, and there was no pulmonary hypertension. CT (computed tomography) angiogram was done for better delineation of anatomy; both echocardiogram and CT revealed a narrowed segment at lower end of RSVC with a diameter of 3 mm and length of 10 mm, while upper part of RSVC had a diameter of 8 mm (Fig. 2). Transcatheter management of SVC obstruction was planned.

**Fig. 3:**
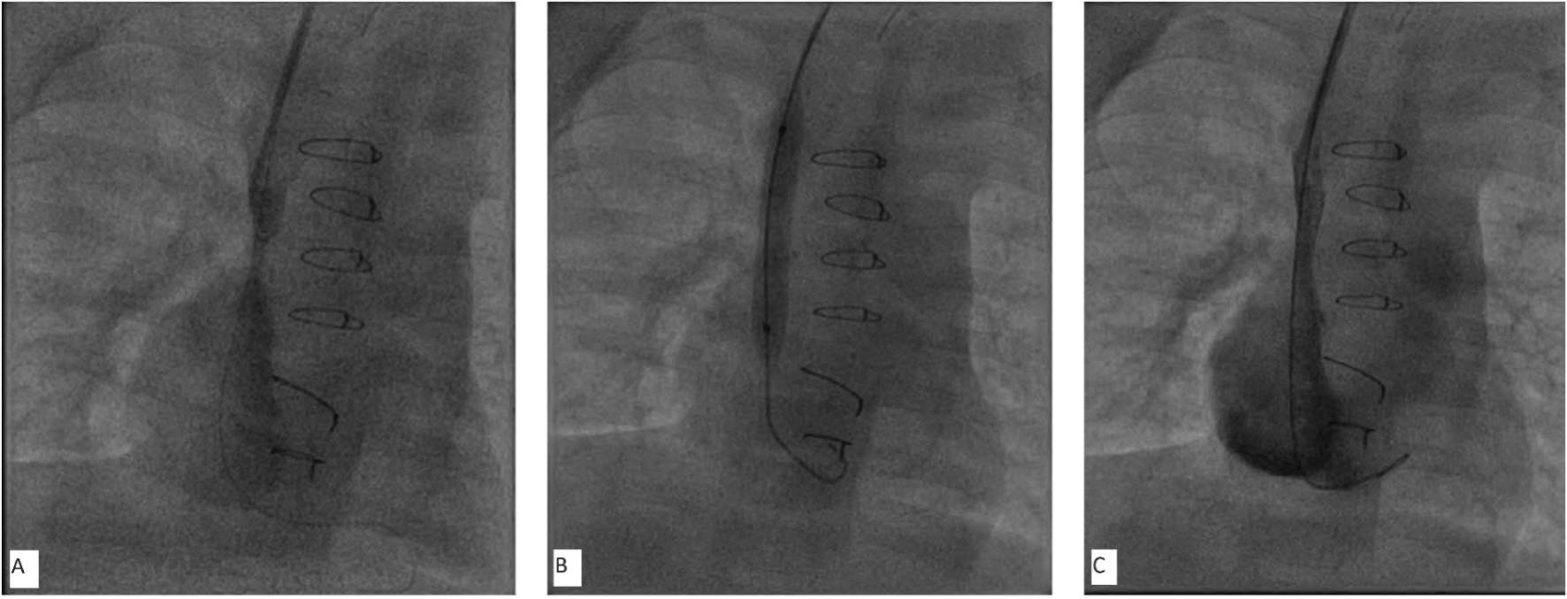
Angiogram in postero-anterior view before balloon angioplasty (**A**), inflation of balloon (**B**) and angiogram after angioplasty showing established flow (**C**)

Cardiac catheterization showed the obstruction with a gradient of 15 mm Hg between upper part of RSVC and RA. Through right internal jugular venous (RIJV) approach, lower end of SVC was dilated using 6 mm × 4 cm Tyshak II balloon catheter (NuMed, Hopkinton, NY) and then by 7 mm × 4 cm Mustang balloon catheter (Boston Scientific, Marlborough, MA) for 15–20 s each time (Fig. 3). Postprocedure pullback gradient across SVC–RA junction came down to 7 mm Hg. The patient was discharged home in a stable condition.

On 1-month follow-up after procedure (body weight 7.6 kg), he again developed signs of SVC obstruction. Transcatheter stenting of SVC was planned. Hemodynamic study showed gradient across lower end of SVC to be 18 mm Hg. Angiogram demonstrated: long tubular narrowing at lower end of SVC measuring 2.8 mm in diameter and 24 mm in length, and SVC diameter superior to narrowing was 7.2 mm. Pulmonary artery angiogram with levophase was done to delineate the right upper pulmonary venous (RUPV) drainage, before and after stenting, to exclude occlusion of RUPV drainage by the stent. Through femoral venous route, 8 mm × 30 mm premounted balloon-expandable bare metal stent (Formula 418, Cook Medical, Bloomington, IN) was placed across lower end of SVC over 0.035″ Amplatz super stiff wire (Cook Medical, Bloomington, IN), with three-fourth of the stent in SVC and one-fourth in atrium. The stent was expanded at 8 atm pressure and lower end flared at 10 atm pressure.

**Fig. 4:**
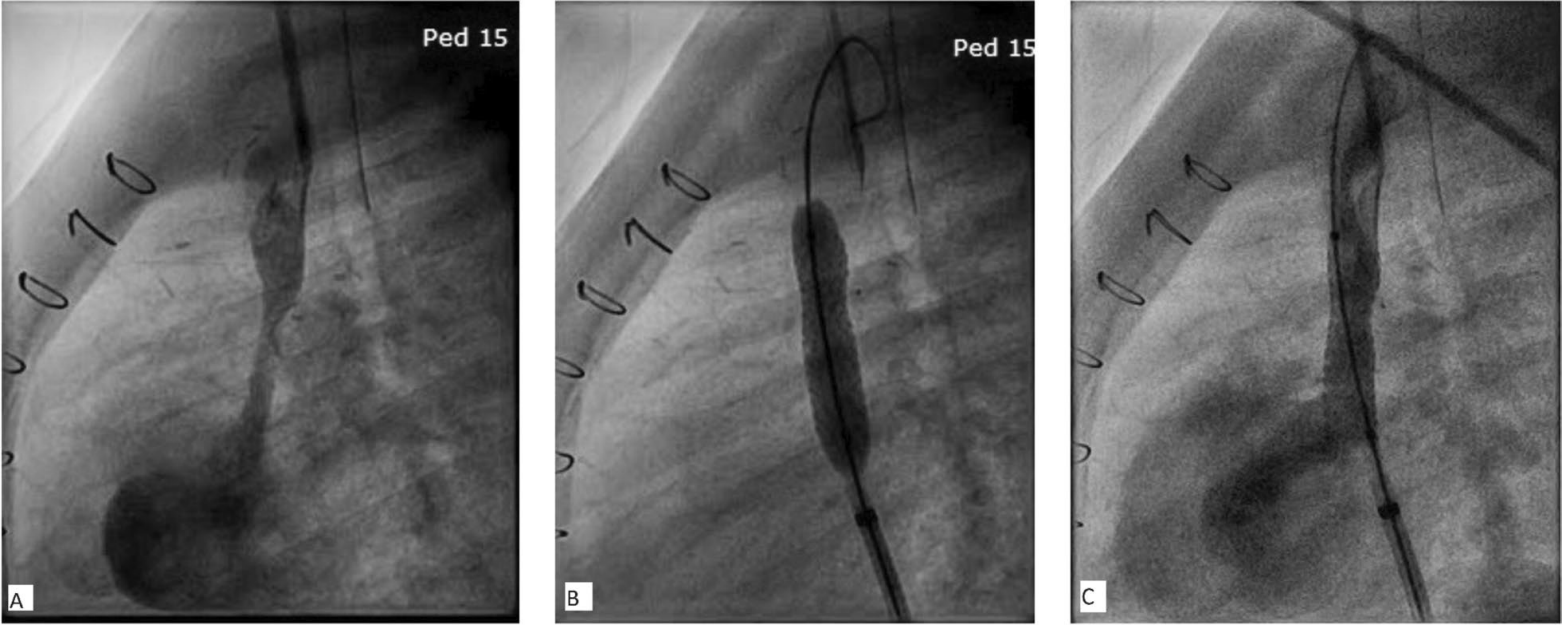
Angiogram in left lateral view before stenting showing narrowed lower part of SVC (**A**), stent inflation (**B**) and angiogram after stent placement showing good flow (**C**)

**Fig. 5:**
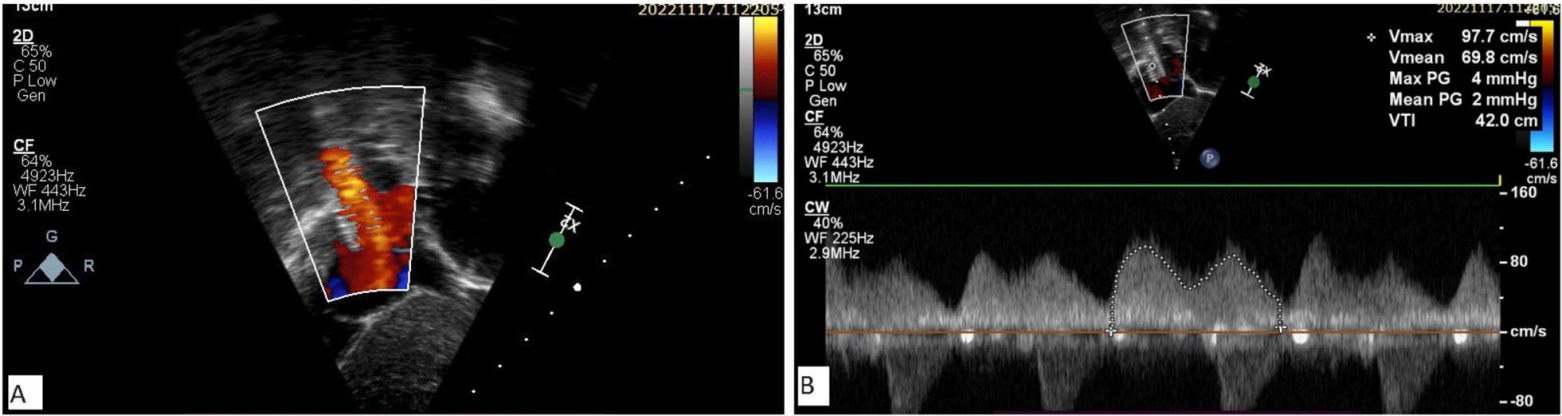
Subcostal bicaval view showing laminar flow across SVC–RA junction (**A**) with mean gradient 2 mm Hg (**B**)

Postprocedure angiogram showed expanded SVC with good flow (Fig. 4). Postprocedure mean gradient on echocardiography came down to 2 mm Hg (Fig. 5). The patient was put on unfractionated heparin infusion for 24 h and discharged home after 2 days on antiplatelet drugs. Follow-up at 1, 3 and 6 months after procedure showed complete disappearance of head and neck swelling, with patent stent flow.

## Discussion

Left untreated, progressive SVC obstruction can lead to grave sequelae like hindered circulation to brain and airway compromise. The relative slow development of narrowing is the reason for absence of symptoms in the immediate postoperative period, as in our case where symptoms were noticed first during follow-up [3]. Transcatheter intervention is preferred over surgical reconstruction due to low procedural morbidity and shorter hospital stay [4]. Endovascular stenting has become first line of management for benign SVC syndrome, providing effective relief of obstruction with less recurrence, as observed in the index case [5]. However, our notion during initial balloon angioplasty was to defer stent placement till the child grows up, which often is the case in small children.

Unlike immediate postoperative situations where choice is limited to covered stents to protect fresh sutures, this case demonstrates bare metal stent to be safe in late postoperative period. The provision of re-dilatation of stent as the child grows contributes to beneficial role of stenting.

Personal communication with surgeon: ASD was closed in this patient with a pericardial patch that extended up to lower end of SVC, and the patch became aneurysmal over time owing to thinned portions of pericardium, causing obstruction. This can be avoided by liberal augmentation of anterior wall of SVC, to allow adequate flow in case patch protrudes from posterior aspect, or using Dacron/polytetrafluoroethylene (PTFE) patch that are expected to behave more predictably and to become less aneurysmal over time.

## Conclusion

Transcatheter stenting can be an effective treatment for postoperative superior vena cava (SVC) stenosis. The index case also underscores importance of close follow-up in these patients.

## Data Availability

The datasets used and/or analyzed during the current study are available from the corresponding author on reasonable request.

## Abbreviations

SVC: Superior vena cava
TAPVC: Total anomalous pulmonary venous connection
RSVC: Right superior vena cava
RA: Right atrium
ASD: Atrial septal defect
CT: Computed tomography
RIJV: Right internal jugular vein
RUPV: Right upper pulmonary vein
Atm: Atmospheric (pressure)
PTFE: Polytetrafluoroethylene
